# Effect of concomitant use of yokukansan on steady‐state blood concentrations of donepezil and risperidone in real‐world clinical practice

**DOI:** 10.1002/npr2.12459

**Published:** 2024-07-08

**Authors:** Junji Saruwatari, Tetsuya Kaneko, Tsukasa Murata, Haruka Narise, Sawa Kugimoto, Eri Nishimura, Natsuki Tetsuka, Misaki Ando, Momo Oi, Masako Ota, Nayumi Hamada, Keiichiro Kaneda, Shiro Furusho, Masakatsu Sakamoto, Ayami Kajiwara‐Morita, Kazutaka Oda, Kentaro Oniki, Keishi Ueda, Hirofumi Jono, Norio Yasui‐Furukori

**Affiliations:** ^1^ Division of Pharmacology and Therapeutics, Graduate School of Pharmaceutical Sciences Kumamoto University Kumamoto Japan; ^2^ Department of Pharmacy Kumamoto University Hospital Kumamoto Japan; ^3^ Kumamoto Seimei Hospital Kumamoto Japan; ^4^ Department of Clinical Pharmaceutical Sciences, Graduate School of Pharmaceutical Sciences Kumamoto University Kumamoto Japan; ^5^ Department of Psychiatry, School of Medicine Dokkyo Medical University Tochigi Japan

**Keywords:** drug interactions, herb‐drug interactions, Kampo, metabolism, pharmacokinetics

## Abstract

**Aim:**

Yokukansan is one of the most frequently used herbal medicines that can improve the behavioral and psychological symptoms of dementia. In this exploratory study, we investigated whether yokukansan affects the steady‐state blood concentrations of donepezil, risperidone, and the major metabolites of both drugs in a real‐world clinical setting.

**Methods:**

A non‐randomized, open‐label, single‐arm study examining drug–drug interactions was conducted. Fifteen dementia patients taking donepezil for at least 4 weeks and eight schizophrenia patients taking risperidone for at least 2 weeks were orally administered 2.5 g of yokukansan three times a day before or between meals, and blood samples were collected before and 8 weeks after starting co‐treatment with yokukansan. Plasma concentrations of donepezil, risperidone, and each metabolite were measured using high‐performance liquid chromatography–tandem mass spectrometry and compared before and after the 8‐week administration of yokukansan.

**Results:**

The plasma concentrations of donepezil and its metabolites (6‐*O*‐desmethyl‐donepezil, 5‐*O*‐desmethyl‐donepezil, and donepezil‐*N*‐oxide), risperidone, and its metabolite paliperidone did not differ before and after the 8‐week treatment with yokukansan.

**Conclusions:**

The findings of this study show that the concomitant use of yokukansan may have little clinical impact on the steady‐state blood levels of donepezil and risperidone in patients with dementia or schizophrenia.

## Abstract 2

This exploratory study investigated whether yokukansan affects steady‐state blood concentrations of donepezil and risperidone, mainly metabolized by CYP2D6 and/or CYP3A, and the major metabolites of both drugs in a real‐world clinical setting. The plasma concentrations of donepezil and its primary metabolites M1, M2, M6, risperidone, its metabolite paliperidone, and the active moiety (i.e., risperidone + paliperidone) did not differ before and after the 8‐week treatment with yokukansan. The findings of this study showed that the effect of yokukansan on steady‐state plasma concentrations of donepezil and risperidone, as well as their metabolites, was small compared with the large individual variability in their blood concentrations.

## Graphical abstract

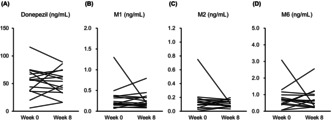

## 1 INTRODUCTION

In Japan, more than 140 Kampo medicines have been approved as prescribed drugs; therefore, they are often used concomitantly with other drugs. Several pharmacodynamic interactions between Kampo medicines and other drugs have been reported. For example, the concomitant use of sho‐saiko‐to and interferon preparations is contraindicated because of the potential development of interstitial pneumonia,^1^ whereas the package insert of other Kampo medicines also cautions that concomitant use of licorice‐containing preparations with loop diuretics or thiazide diuretics increases the risk of pseudoaldosteronism and that concomitant use of ephedra‐containing preparations with MAO inhibitors increases the sympathomimetic effect of these drugs.^1^ On the other hand, there is little information on the pharmacokinetic interactions of Kampo medicines. Since Kampo medicines are mixtures of several herbal medicines, there is a possibility that the effects of each component in the body may be more than mere addition and subtraction or that they may have effects that do not appear alone. Therefore, conducting clinical trials on human subjects to investigate drug–drug interactions with Kampo medicines is clinically important. Based on the information noted above, we have previously studied the effects of several Kampo medicines on the activity of drug‐metabolizing enzymes, such as cytochrome P450 (CYP), in healthy adult subjects with the aim of predicting pharmacokinetic interactions between Kampo medicines and other prescribed drugs.^2^, ^3^, ^4^

Symptoms of dementia include core symptoms, such as memory impairment and disorientation, and Behavioral and Psychological Symptoms of Dementia (BPSD), such as delusions and irritability.^5^ Since BPSD occurs in over one‐third of individuals with dementia and is associated with worsening activities of daily living, faster cognitive decline, and a poorer quality of life for individuals with dementia,^5^ it is essential to address BPSD when treating dementia. Yokukansan, one of the most frequently used Kampo medicines, has been attracting attention as a treatment for BPSD, and the results of a randomized, blinded, controlled trial in dementia patients with BPSD showed that patients treated with yokukansan significantly improved their Neuropsychiatric Inventory total score, an evaluation scale for BPSD, and their Barthel Index Score, a measure of daily activities of living,^6^ and the efficacy of the medicine was also confirmed in other trials.^7^, ^8^, ^9^

In our previous study, 7‐day administration of yokukansan (5 g/day) did not affect the in vivo activity of CYP3A, CYP2D6, and CYP1A2 in healthy young male subjects,^4^ suggesting that yokukansan is unlikely to interact with drugs that are metabolized by these enzymes. However, the activity of drug‐metabolizing enzymes is influenced by age, sex, genetic background, and underlying disease,^10^, ^11^ and it is possible that these patient factors may also influence the extent of drug interactions.^12^, ^13^ In particular, yokukansan is often administered to elderly patients in clinical practice. The disposition of the drug in the elderly may differ from that in healthy adults due to age‐related physiological changes, such as a decreased hepatic metabolic clearance,^14^, ^15^ thus, the effects of yokukansan administration on the drug‐metabolizing enzymes of young and elderly individuals may be different. Therefore, to demonstrate the clinical impact of yokukansan on drug‐metabolizing enzyme activity, it is necessary to examine the changes in the disposition of drugs metabolized by each enzyme (e.g., drug concentrations in blood) under clinically relevant conditions in a patient population, such as dementia patients.

Donepezil is primarily metabolized by CYP3A and CYP2D6,^16^ and risperidone is predominantly metabolized by CYP2D6 (and some CYP3A).^17^ Large variability in the drug response to donepezil and risperidone has been reported in patients with dementia and schizophrenia, respectively, which might partly be due to inter‐individual differences in plasma concentrations of the drugs.^18^, ^19^ Against this background, the primary objective of this exploratory study was to determine whether yokukansan affects the steady‐state plasma concentrations of donepezil, risperidone, and the primary metabolites of both drugs in a real‐world clinical setting.

## 2 METHODS

This was a non‐randomized, open‐label, single‐arm study that examined drug–drug interactions. Patients with dementia who had been taking donepezil hydrochloride for at least 4 weeks and patients with schizophrenia who had been taking risperidone for at least 2 weeks were enrolled in this clinical study. Among the enrolled patients, those who initiated the concomitant use of yokukansan, received oral yokukansan (2.5 g) three times a day before or between meals, and agreed to participate were included in the present study. The Data S1 section presents a detailed protocol.

Plasma concentrations of donepezil and its primary metabolites, 6‐*O*‐demsmethyl‐donepezil (M1), 5‐*O*‐demsmethyl‐donepezil (M2), donepezil‐*N*‐oxide (M6), risperidone, and its major metabolite, 9‐hydroxyrisperidone (paliperidone), were determined using a liquid‐chromatograph‐tandem mass spectrometer with some modifications of the method reported by Noetzli et al.^20^ and Yasui‐Furukori et al.^21^ DNA was extracted from a peripheral blood sample. The presence or absence of the CYP2D6*5, CYP2D6*10, CYP3A5*3, and P450 oxidoreductase (POR)*28 polymorphisms was determined. The detailed analytical procedures are provided in Data S1.

Geometric mean ratios and 90% confidence intervals (CIs) were calculated for each blood concentration based on the values before (week 0) and after the 8‐week concomitant use of yokukansan (week 8). Blood drug concentrations at weeks 0 and 8 were compared using a paired *t*‐test and a repeated measures analysis of variance, considering the interaction between genotype and the concomitant use of yokukansan. Statistical significance was set at *p* < 0.05. Multiple comparisons were corrected using Bonferroni's method, and *p*‐values < 0.05/*n* were considered statistically significant after correcting for the number of comparisons. All statistical analyses were performed using R software (ver. 4.0.5; R Foundation for Statistical Computing, Vienna, Austria).

## 3 RESULTS

**FIGURE 1:**
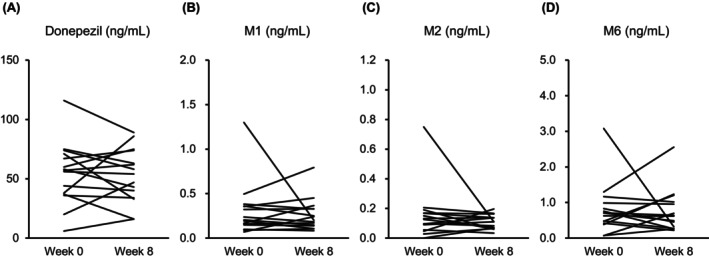
Individual values of plasma concentrations of donepezil (A) and its major metabolites M1 (B), M2 (C), and M6 (D) before (week 0) and after (week 8) the concomitant use of yokukansan.

**FIGURE 2:**
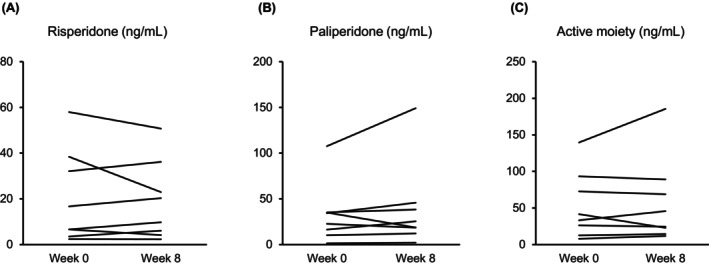
Individual values of plasma concentrations of risperidone (A), its major metabolite paliperidone (B), and the active moiety (C) before (week 0) and after (week 8) the concomitant use of yokukansan.

Fifteen dementia patients taking 5 mg donepezil hydrochloride and eight schizophrenia patients taking 1–8 mg risperidone oral disintegrating tablets were included in this study (Table S1). All subjects were not taking any concomitant medications that affected the disposition of donepezil or risperidone before and during the period of concomitant use of yokukansan. There were no statistically significant differences in the plasma concentrations of donepezil and its primary metabolites M1, M2, M6, risperidone, its metabolite paliperidone, and the active moiety (i.e., risperidone + paliperidone) at weeks 0 and 8 (Tables 1 and 2, Figures 1 and 2).

**TABLE 1:** Comparison of blood levels of donepezil and its major metabolites M1, M2, and M6 before (week 0) and after (week 8) the concomitant use of yokukansan.

	Unit	Week	Mean	Standard deviation	Week 8/week 0^a^ (90% confidence interval)	*p* Value^b^
Donepezil	ng/mL	0	54.33	26.23	1.02 (0.80–1.30)	0.772
		8	52.67	22.87		
M1	ng/mL	0	0.305	0.302	0.95 (0.69–1.29)	0.577
		8	0.259	0.182		
M2	ng/mL	0	0.154	0.176	0.82 (0.55–1.22)	0.323
		8	0.107	0.048		
M6	ng/mL	0	0.803	0.720	0.88 (0.56–1.38)	0.810
		8	0.746	0.609		

^a^ Ratio of the geometric mean at week 8 to that at week 0 for each plasma concentration.

^b^ Plasma concentrations at weeks 0 and 8 were compared using paired *t*‐test.

**TABLE 2:** Comparison of blood concentrations of risperidone, its major metabolite paliperidone, and active moiety before (week 0) and after (week 8) the concomitant use of yokukansan.

	Unit	Week	Mean	Standard deviation	Week 8/week 0^a^ (90% confidence interval)	*p* Value^b^
Risperidone	ng/mL	0	20.55	20.29	1.01 (0.78–1.30)	0.553
		8	19.04	17.22		
Paliperidone	ng/mL	0	32.81	32.69	1.12 (0.88–1.44)	0.351
		8	38.72	46.77		
Active moiety [risperidone + paliperidone]	ng/mL	0	53.35	45.41	1.05 (0.84–1.30)	0.531
		8	57.76	58.39		

^a^ Ratio of the geometric mean at week 8 to that at week 0 for each plasma concentration.

^b^ Plasma concentrations at weeks 0 and 8 were compared using paired *t*‐test.

The genotype frequencies of CYP2D6*10, CYP3A5*3, and POR*28 polymorphisms in 15 patients treated with donepezil and the eight patients taking risperidone were shown in Table S1. CYP2D6*5 was not detected in any of the subjects. Genetic polymorphisms and concomitant use of yokukansan had no interactive effects on the plasma concentrations of donepezil, risperidone, or their metabolites (Tables S2 and S3, Figures S1–S6).

## 4 DISCUSSION

In this study, the blood levels of donepezil and risperidone, which are mainly metabolized by CYP2D6 and/or CYP3A, and the primary metabolites of both drugs were compared before and after an 8‐week treatment with yokukansan (7.5 g/day) in patients with dementia and schizophrenia. No significant differences were found in any of the blood levels before or after the use of yokukansan.

We previously studied the effects of repeated 7‐day doses of 5.0 g/day yokukansan on CYP3A and CYP2D6 enzyme activities in healthy young male subjects and reported no significant impact on either activity.^4^ However, as noted above, yokukansan is often administered to older people in clinical practice. It has been reported that inhibition of oxycodone metabolism via CYP3A by clarithromycin was similar in young and elderly patients.^22^ Meanwhile, the induction of CYP3A‐mediated antipyrine metabolism by rifampicin was lower in the elderly than in the young.^23^ The results of this study suggest that the concomitant use of yokukansan may have little clinical impact on the blood levels of donepezil and risperidone to affect the significant intra‐patient differences in the disposition of both drugs in a clinical setting.

CYP2D6 metabolizes many psychiatric drugs, including antipsychotics and anti‐dementia drugs. Yokukansan consists of seven herbs (i.e., *Atractylodis lanceae* rhizoma, *Poria sclerotium*, *Cnidium* rhizome, *Uncaria* hook, Japanese *Angelica* root, *Bupleurum* root, and *Glycyrrhiza*), and liquiritigenin, a flavonoid contained in *Glycyrrhiza*, was reported to have CYP2D6 inhibitory activity in human liver microsomes.^24^ Meanwhile, CYP3A is involved in metabolizing ~30% of drugs.^10^ In vitro studies have reported that the *Glycyrrhiza* root and its constituent, vestitol, in yokukansan have CYP3A4 inhibitory activity.^25^ Meanwhile, glycyrrhizin, a component contained in *Glycyrrhiza* root, was reported to induce CYP3A.^26^, ^27^ The present findings and our previous results^4^ suggest that the influence of yokukansan on the dispositions of donepezil and risperidone, especially the metabolic processes, is low in humans in vivo.

Significant individual differences exist in the degree of inhibition of enzymes such as CYP by drugs, and this is the essential factor that explains the individual differences in CYP activity before drug administration.^12^ For example, *CYP2D6* polymorphisms are known to contribute significantly to individual differences in CYP2D6 activity,^10^ and it is recommended that genetic polymorphisms be considered in studies of drug–drug interactions.^13^ Meanwhile, POR is an electron‐transfer cofactor for all CYP enzymes and some non‐P450 enzymes (e.g., cytochrome b5) in microsomes.^28^, ^29^ POR*28 (c.1508C>T, rs1057868) is a common polymorphism in various ethnic groups,^28^, ^30^ and the metabolic activity and plasma metabolite concentrations of midazolam, which CYP3A primarily metabolizes, are reported to be significantly higher in holders of the T/T genotype of POR*28 than in holders of the C allele.^10^, ^29^ In this study, we determined the CYP2D6*10, CYP3A5*3, and POR*28 polymorphisms, which are more prevalent in East Asians and which impact CYP2D6 or CYP3A activity, and found no significant interaction between the polymorphisms and yokukansan co‐treatment with regard to blood levels of donepezil, risperidone, and their metabolites. Therefore, even when these genetic polymorphisms are considered, yokukansan may have a negligible effect on the pharmacokinetics of both drugs.

This exploratory study's design was observational to best reflect the actual clinical situation. However, this needs to be validated in a larger population because the number of subjects in our study was small. Although the differences in the plasma concentrations at week 0 and week 8 did not reach statistical significance, the 90% CIs of the geometric mean ratio (week 8 to week 0) were outside the bioequivalence range of 0.80–1.25.^13^ If we simply estimated based on the results, such as means and standard deviations, observed in this study, 10 and 41 patients were supposed to be sufficient to detect a 50% difference in the steady‐state blood levels of donepezil and risperidone, respectively, between week 0 and week 8, with a power of 0.80 (α‐level of 0.05). Furthermore, since the statistical analyses in the present study did not consider other patient factors (e.g., age, sex, drug dosage, concomitant medications, hepatic function, and renal function), except for genotypes, we believe that future extensive studies incorporating more detailed information will prove whether donepezil or risperidone can be safely used with yokukansan.

Despite the limitations stated above, the findings of this study showed that the effect of yokukansan on steady‐state plasma concentrations of donepezil, risperidone, and their metabolites was small in comparison to the large individual variability in their blood concentrations, and the risk of pharmacokinetic interactions with donepezil or risperidone was considered to be low. Further investigations with larger numbers of patients are needed to verify the influence of yokukansan on the pharmacokinetics and pharmacodynamics of donepezil and risperidone.

## AUTHOR CONTRIBUTIONS

JS, TK, AK‐M, KOniki, HJ, and NY‐F analyzed the data and wrote the first draft of the manuscript. TK, HN, SK, EN, NT, MA, MOi, MOta, AK‐M, KOda, and KOniki measured blood drug/metabolite concentrations and genotyping. TM, NH, KK, SF, MS, and NY‐F collected samples. All authors contributed substantially to the study design and approved the final manuscript.

## FUNDING INFORMATION

This study was supported by a research grant from Tsumura & Co. The funder played no role in the study design, data collection, analysis, manuscript writing, and publication decisions.

## CONFLICT OF INTEREST STATEMENT

Norio Yasui‐Furukori and Junji Saruwatari are Editorial Board members of Neuropsychopharmacology Reports and co‐authors of this article. To minimize bias, they were excluded from all editorial decision‐making related to the acceptance of this article for publication. Junji Saruwatari and Hirofumi Jono were supported by the research grants from Tsumura & Co. The remaining authors declare no conflict of interest.

## ETHICS STATEMENT

Approval of the Research Protocol by an Institutional Reviewer Board: The study protocol was approved by the Ethics Committee of the Faculty of Life Sciences, Kumamoto University. (Approval No. Genome 363). This protocol was conducted in accordance with the principles of the Declaration of Helsinki and the Japanese Ethical Guidelines for Medical and Health Research Involving Human Subjects.

Informed Consent: Informed consent was obtained from all subjects involved in the study, and written informed consent was obtained from all patients for the publication of this paper.

Registry and the Registration No. of the Study/Trial: UMIN000026273.

Animal Studies: N/A.

## Supporting information


Data S1.

Figure S1.
Figure S2.
Figure S3.
Figure S4.
Figure S5.
Figure S6.

Table S1.
Table S2.
Table S3.

## Data Availability

The data, including genetic information, of the present study were not made publicly available because the disclosure of individual data was not included in the research protocol, and consent for public data sharing was not obtained from the participants.

## References

[1] Shimada Y . Adverse effects of Kampo medicines. Intern Med. 2022;61(1):29–35. 33551404 10.2169/internalmedicine.6292-20 PMC8810258

[2] Saruwatari J , Nakagawa K , Shindo J , Nachi S , Echizen H , Ishizaki T . The in‐vivo effects of sho‐saiko‐to, a traditional Chinese herbal medicine, on two cytochrome P450 enzymes (1A2 and 3A) and xanthine oxidase in man. J Pharm Pharmacol. 2003;55(11):1553–1559. 14713367 10.1211/0022357022061

[3] Saruwatari J , Takaishi C , Yoshida K , Takashima A , Fujimura Y , Umemoto Y , et al. A herbal‐drug interaction study of keishi‐bukuryo‐gan, a traditional herbal preparation used for menopausal symptoms, in healthy female volunteers. J Pharm Pharmacol. 2012;64(5):670–676. 22471362 10.1111/j.2042-7158.2011.01443.x

[4] Soraoka H , Oniki K , Matsuda K , Ono T , Taharazako K , Uchiyashiki Y , et al. The effect of Yokukansan, a traditional herbal preparation used for the behavioral and psychological symptoms of dementia, on the drug‐metabolizing enzyme activities in healthy male volunteers. Biol Pharm Bull. 2016;39(9):1468–1474. 27582327 10.1248/bpb.b16-00248

[5] Tampi RR , Bhattacharya G , Marpuri P . Managing behavioral and psychological symptoms of dementia (BPSD) in the era of boxed warnings. Curr Psychiatry Rep. 2022;24(9):431–440. 35781675 10.1007/s11920-022-01347-y

[6] Iwasaki K , Satoh‐Nakagawa T , Maruyama M , Monma Y , Nemoto M , Tomita N , et al. A randomized, observer‐blind, controlled trial of the traditional Chinese medicine Yi‐Gan san for improvement of behavioral and psychological symptoms and activities of daily living in dementia patients. J Clin Psychiatry. 2005;66(2):248–252. 15705012 10.4088/jcp.v66n0214

[7] Mizukami K , Asada T , Kinoshita T , Tanaka K , Sonohara K , Nakai R , et al. A randomized cross‐over study of a traditional Japanese medicine (Kampo), yokukansan, in the treatment of the behavioural and psychological symptoms of dementia. Int J Neuropsychopharmacol. 2009;12(2):191–199. 19079814 10.1017/S146114570800970X

[8] Monji A , Takita M , Samejima T , Takaishi T , Hashimoto K , Matsunaga H , et al. Effect of yokukansan on the behavioral and psychological symptoms of dementia in elderly patients with Alzheimer's disease. Prog Neuropsychopharmacol Biol Psychiatry. 2009;33(2):308–311. 19138715 10.1016/j.pnpbp.2008.12.008

[9] Teranishi M , Kurita M , Nishino S , Takeyoshi K , Numata Y , Sato T , et al. Efficacy and tolerability of risperidone, yokukansan, and fluvoxamine for the treatment of behavioral and psychological symptoms of dementia: a blinded, randomized trial. J Clin Psychopharmacol. 2013;33(5):600–607. 23948783 10.1097/JCP.0b013e31829798d5

[10] Zanger UM , Schwab M . Cytochrome P450 enzymes in drug metabolism: regulation of gene expression, enzyme activities, and impact of genetic variation. Pharmacol Ther. 2013;138(1):103–141. 23333322 10.1016/j.pharmthera.2012.12.007

[11] Chothe PP , Arya V , Prasad B , Ramsden D , Taskar K . Innovations, opportunities, and challenges for predicting alteration in drug‐metabolizing enzyme and transporter activity in specific populations. Drug Metab Dispos. 2023;51(12):1547–1550. 37775331 10.1124/dmd.123.001453 PMC10658904

[12] Viviani R , Berres J , Stingl JC . Phenotypic models of drug‐drug‐gene interactions mediated by cytochrome drug‐metabolizing enzymes. Clin Pharmacol Ther. 2024. in press. 10.1002/cpt.3188 38318716

[13] Pharmaceuticals and Medical Devices Agencies . Guideline on drug interaction for drug development and appropriate provision of information [Internet]. Tokyo: Pharmaceuticals and Medical Devices Agencies; 2019. [cited 8 Feb 2019]. Available from: https://www.pmda.go.jp/files/000228122.pdf

[14] Klotz U . Pharmacokinetics and drug metabolism in the elderly. Drug Metab Rev. 2009;41(2):67–76. 19514965 10.1080/03602530902722679

[15] Soejima K , Sato H , Hisaka A . Age‐related change in hepatic clearance inferred from multiple population pharmacokinetic studies: comparison with renal clearance and their associations with organ weight and blood flow. Clin Pharmacokinet. 2022;61(2):295–305. 34514537 10.1007/s40262-021-01069-z

[16] Noetzli M , Eap CB . Pharmacodynamic, pharmacokinetic and pharmacogenetic aspects of drugs used in the treatment of Alzheimer's disease. Clin Pharmacokinet. 2013;52(4):225–241. 23408070 10.1007/s40262-013-0038-9

[17] Zhang L , Brown SJ , Shan Y , Lee AM , Allen JD , Eum S , et al. CYP2D6 genetic polymorphisms and risperidone pharmacokinetics: a systematic review and meta‐analysis. Pharmacotherapy. 2020;40(7):632–647. 32519344 10.1002/phar.2434

[18] Coin A , Pamio MV , Alexopoulos C , Granziera S , Groppa F , de Rosa G , et al. Donepezil plasma concentrations, CYP2D6 and CYP3A4 phenotypes, and cognitive outcome in Alzheimer's disease. Eur J Clin Pharmacol. 2016;72(6):711–717. 26952092 10.1007/s00228-016-2033-1

[19] Li HQ , Xu JY , Gao YY , Jin L . Optimization of maintenance therapy of risperidone with CYP2D6 genetic polymorphisms through an extended translational framework‐based prediction of target occupancies/clinical outcomes. Pharmacol Res. 2018;137:135–147. 30281999 10.1016/j.phrs.2018.09.030

[20] Noetzli M , Choong E , Ansermot N , Eap CB . Simultaneous determination of antidementia drugs in human plasma for therapeutic drug monitoring. Ther Drug Monit. 2011;33(2):227–238. 21383648 10.1097/FTD.0b013e31821126cf

[21] Yasui‐Furukori N , Mihara K , Takahata T , Suzuki A , Nakagami T , de Vries R , et al. Effects of various factors on steady‐state plasma concentrations of risperidone and 9‐hydroxyrisperidone: lack of impact of MDR‐1 genotypes. Br J Clin Pharmacol. 2004;57(5):569–575. 15089809 10.1111/j.1365-2125.2003.02061.x PMC1884506

[22] Liukas A , Hagelberg NM , Kuusniemi K , Neuvonen PJ , Olkkola KT . Inhibition of cytochrome P450 3A by clarithromycin uniformly affects the pharmacokinetics and pharmacodynamics of oxycodone in young and elderly volunteers. J Clin Psychopharmacol. 2011;31(3):302–308. 21508859 10.1097/JCP.0b013e3182189892

[23] Twum‐Barima Y , Finnigan T , Habash AI , Cape RD , Carruthers SG . Impaired enzyme induction by rifampicin in the elderly. Br J Clin Pharmacol. 1984;17(5):595–597. PMC1463462 6733008

[24] Qiao X , Ji S , Yu SW , Lin XH , Jin HW , Duan YK , et al. Identification of key licorice constituents which interact with cytochrome P450: evaluation by LC/MS/MS cocktail assay and metabolic profiling. AAPS J. 2014;16(1):101–113. 24254844 10.1208/s12248-013-9544-9 PMC3889530

[25] Tsukamoto S , Aburatani M , Yoshida T , Yamashita Y , El‐Beih AA , Ohta T . CYP3A4 inhibitors isolated from licorice. Biol Pharm Bull. 2005;28(10):2000–2002. 16204965 10.1248/bpb.28.2000

[26] Wang YG , Zhou JM , Ma ZC , Li H , Liang QD , Tan HL , et al. Pregnane X receptor mediated‐transcription regulation of CYP3A by glycyrrhizin: a possible mechanism for its hepatoprotective property against lithocholic acid‐induced injury. Chem Biol Interact. 2012;200(1):11–20. 22982774 10.1016/j.cbi.2012.08.023

[27] Tu JH , He YJ , Chen Y , Fan L , Zhang W , Tan ZR , et al. Effect of glycyrrhizin on the activity of CYP3A enzyme in humans. Eur J Clin Pharmacol. 2010;66(8):805–810. 20393696 10.1007/s00228-010-0814-5

[28] Huang N , Agrawal V , Giacomini KM , Miller WL . Genetics of P450 oxidoreductase: sequence variation in 842 individuals of four ethnicities and activities of 15 missense mutations. Proc Natl Acad Sci USA. 2008;105(5):1733–1738. 18230729 10.1073/pnas.0711621105 PMC2234213

[29] Miller WL , Agrawal V , Sandee D , Tee MK , Huang N , Choi JH , et al. Consequences of POR mutations and polymorphisms. Mol Cell Endocrinol. 2011;336(1–2):174–179. 21070833 10.1016/j.mce.2010.10.022 PMC4632974

[30] Saito Y , Yamamoto N , Katori N , Maekawa K , Fukushima‐Uesaka H , Sugimoto D , et al. Genetic polymorphisms and haplotypes of por, encoding cytochrome p450 oxidoreductase, in a Japanese population. Drug Metab Pharmacokinet. 2011;26(1):107–116. 21084761 10.2133/dmpk.dmpk-10-sc-096

