# Supplementary material for: Effect of concomitant use of yokukansan on steady‐state blood concentrations of donepezil and risperidone in real‐world clinical practice
Source: Neuropsychopharmacol Rep. 2024 Jul 8;44(3):614–9. doi: 10.1002/npr2.12459 (PMC11544453; doi:10.1002/npr2.12459)
Supplement: Supplementary file 1 — Data S1. [file NPR2-44-614-s002.docx]

**Supplementary Methods**

**1 Study Protocol**

*1.1 Study design*

The study design was a non-randomized, open-label, single-arm study (UMIN000026273) examining drug-drug interactions (Period of Study Implementation, December 25, 2017–March 31, 2024).

*1.2 Study subjects*

Patients treated at Dokkyo Medical University Hospital, Hirosaki University Hospital, and Kumamoto Seimei Hospital and met the following eligibility criteria and did not meet the exclusion criteria were included:

*Eligibility criteria*

1) Patients diagnosed with schizophrenia or dementia according to the Diagnostic and Statistical Manual of Mental Disorders, fifth edition, or the International Classification of Disease, tenth revision.

2) Patients with dementia taking donepezil hydrochloride for at least four weeks or patients with schizophrenia taking risperidone for at least two weeks.

3) Patients of ≥20 years of age.

4) Patients who do not have diseases that affect drug metabolism and excretion (e.g., gastrointestinal tract, heart, lung, liver, or congenital metabolic disorders).

5) Patients who, after an adequate explanation, have given written informed consent to participate in this study

*Exclusion criteria*

1. Patients who used yokukansan or other herbal medicines within two weeks before enrollment in the present study.
2. Patients who received medications that affect the dispositions of donepezil and risperidone within four weeks before enrollment in the present study.
3. Patients who used any investigational drugs within three months before enrollment in the present study.

*1.3 Drugs used in the study*

1) Tsumura Yokukansan Extract Granules (for medical use) 7.5 g/day (3 times a day).

2) Donepezil hydrochloride OD tablet 5 mg.

3) Risperidone tablets 1mg, Risperidone tablets 2mg, Risperidone tablets 3mg.

Yokukansan (2.5 g) was orally administered three times daily before or between meals. Compliance with the yokukansan was assessed using a pill count by a hospital pharmacist and a self-report of missed doses during the study period.

*1.4 Sample collection*

Blood samples were collected before, four weeks after, and eight weeks after the administration of yokukansan. Whole blood samples (15 mL) were collected before and eight weeks after starting the administration of yokukansan. After centrifugation of the peripheral blood collected in EDTA-2Na collection tubes, the plasma portion was used for blood drug concentration measurements, and the rest was used for genotyping.

*1.5 Other information collected*

The following information was also obtained from the medical records:

Sex, age, weight, dose of the study drug (donepezil hydrochloride or risperidone), time of administration, time of blood collection, other concomitant medications, underlying and comorbid diseases, smoking history, and alcohol consumption.

*1.6 Primary endpoints*

The primary endpoints were 1) the changes in the plasma concentrations of donepezil and its metabolites before and after the concomitant use of yokukansan and 2) the change in the plasma concentrations of risperidone and its metabolites before and after the concomitant use of yokukansan.

*1.6 Secondary endpoints*

The secondary endpoints were the effect of genetic polymorphisms on the plasma concentrations of donepezil, risperidone, and their metabolites before and after the concomitant use of yokukansan.

**2 Determination of Plasma Concentrations of Donepezil and Its Metabolites**

*2.1 Calibration curve*

First, stock solutions of donepezil, M1, M2, M6, and internal standards (Donepezil-d4 (D-d4), 6-o-Desmethyl Donepezil-d5 (M1-d5), 5-o-Desmethyl Donepezil-d5 (M2-d5)) were prepared. Then, the stock solutions of donepezil, M1, M2, and M6 were dissolved in blank human plasma (plasma samples from patients not taking donepezil) to concentrations of 50, 0.5, and 0.5 ng/mL, respectively (100 μL).

*2.2 Liquid chromatograph-tandem mass spectrometer (LC/MS/MS) System*

The LC/MS/MS system was a SHIMADZU LC/MS/MS system LCMS-8040, and the analytical column was a Cadenza CD-C18 column. MS was performed in the proton precursor ion [M + H] mode.

*2.3 Preparation of plasma samples*

A solid-phase extraction column (Waters Oasis HLB 1 cc, 30 mg, 30 μm, 100 pk, stabilized with 1 mL of 100% methanol and 1 mL of distilled water) was used to wash 100 μL of plasma sample and ten μL of internal standard with 1000 μL of distilled water (three times) and 0.5 mL of 10% methanol. The samples were eluted with 1 mL 100% methanol. The solvent was then removed using an evaporator (40°C), and the residue was redissolved in 50 µL of the mobile phase.

*2.4 Mobile phase*

Thirty milliliters and 70 mL of (A) and (B) were mixed.

(A) Methanol: 100 mL Ammonium acetate: 77 mg Acetic acid: 100 mL

(B) Water: 100 mL Ammonium acetate: 77 mg Acetic acid: 100 mL

*2.5 Validation of the measurement method*

The degree of linearity compliance was considered valid when the square of the correlation coefficient (r) for the concentration range of the calibration curve was ≥0.98. Samples for the calibration curve were measured repeatedly (10 times), and when both the coefficient of variation and mean absolute error were <15%, the method was judged to be reproducible.

**3. Determination of Plasma Concentrations of Risperidone and Its Metabolite Paliperidone**

*3.1 Calibration curve*

First, stock solutions of risperidone and paliperidone were prepared and dissolved in methanol to obtain 3.75 to 250 ng/mL. An internal standard risperidone-d4 solution was prepared and dissolved in methanol to obtain ten ng/mL. Samples for the calibration curve were prepared by mixing 400 μL of blank human plasma samples from patients who were not taking risperidone with 100 μL of the above mixture of risperidone and paliperidone to obtain the calibration range from 0.75 to 50 ng/mL.

*3.2 LC/MS/MS System*

The LC/MS/MS system was an LCMS-8040 (SHIMADZU CORPORATION, Kyoto, Japan), and the analytical column was a HYPERSIL BDS C18 column (pore size of 5 μm, 3 mm×100 mm, Thermo Fisher Scientific, Waltham, USA), the MS conditions were proton precursor ion [M+H] mode in electrospray ionization. The analyte-specific m/z values for precursor and corresponding fragment ions were 411.3/191.2 for risperidone, 427.15/207.20 for paliperidone, and 415.15/195.3 for risperidone d4, respectively.

*3.3 Preparation of plasma samples*

As the pretreatment of the samples, 1,800 μL of 0.05 mol/L ammonium acetate and 100 μL of the internal standard solution were first added. Solid-phase extraction columns Biotage 601-0001-AXG EVOLUTE EXPRESS CX 10 mg/1mL (Tabless) were activated with 3 mL of 100% methanol and subsequent 3 mL of 0.05 mol/L ammonium acetate (pH=6) 3 mL. The pretreated samples were loaded onto the SPE columns and then washed with 500 μL of 0.05 mol/L ammonium acetate (pH=6) and 500 μL of 100% methanol. The target compounds were eluted with 200 μL of methanol/ammonium hydroxide (95:5, v/v). The solvent was then removed by a vacuum decompression evaporator (40°C) for 30 min, and the residue was redissolved in 120 μL of the mobile phase.

*3.4 Mobile phase*

(A), (B) and (C) were used as the washing solutions.

(A) 0.01 M ammonium formate (pH=4.0)

(B) 100% acetonitrile

(C) 100% methanol

*3.5 Validation of the measurement method*

The degree of linearity compliance was considered valid when the square of the correlation coefficient (r) was 0.98 or greater in relation to the concentration range of the calibration curve. Samples for the calibration curve were measured repeatedly (10 times), and when both the coefficient of variation and mean absolute error were <5%, the method was judged to be reproducible.

**4 Genotyping**

Peripheral blood from which the plasma was separated was subjected to DNA extraction using the Flexi Gene DNA kit (Qiagen, Hilden, Germany). DNA was extracted according to the manufacturer’s instructions. The *CYP2D6*5*, *CYP2D6*10*, *CYP3A5*3*, and *POR*28* genes, which are known to affect the activity of CYP2D6 and CYP3A, the main metabolic enzymes in the metabolism of donepezil and risperidone, respectively, were analyzed for the presence or absence of the *POR*28* gene polymorphisms as described in our previous reports.^1-4^ Specifically, the *CYP2D6*5* polymorphism was determined by polymerase chain reaction (PCR), and the *CYP2D6*10*, *CYP3A5*3*, and *POR*28* polymorphisms were determined by TaqMan PCR using commercially available primers and probes (nucleotide sequences not disclosed) in a real-time PCR Step One Plus Real-Time PCR system (version 2.1, Applied Biosystems, Tokyo, Japan). *CYP2D6 non-*10*, *CYP3A5 non-*3*, and *POR non-*28* were the ‘default’ assignments if no variation was found.

**REFERENCES**

1. Soraoka H, Oniki K, Matsuda K, et al. The Effect of Yokukansan, a Traditional Herbal Preparation Used for the Behavioral and Psychological Symptoms of Dementia, on the Drug-Metabolizing Enzyme Activities in Healthy Male Volunteers. Biol Pharm Bull. 2016;39(9):1468-74.

2. Saruwatari J, Ogusu N, Shimomasuda M, et al. Effects of CYP2C19 and P450 oxidoreductase polymorphisms on the population pharmacokinetics of clobazam and N-desmethylclobazam in Japanese patients with epilepsy. Ther Drug Monit. 2014;36(3):302-9.

3. Nishimura M, Ueda M, Saruwatari J, et al. Influence of the cytochrome P450 2D6 *10/*10 genotype on the pharmacokinetics of paroxetine in Japanese patients with major depressive disorder: a population pharmacokinetic analysis. Pharmacogenet Genomics. 2016;26(9):403-13.

4. Saruwatari J, Yoshida S, Tsuda Y, et al. Pregnane X receptor and hepatocyte nuclear factor 4alpha polymorphisms are cooperatively associated with carbamazepine autoinduction. Pharmacogenet Genomics. 2014;24(3):162-71.
