# Supplementary material for: Effect of concomitant use of yokukansan on steady‐state blood concentrations of donepezil and risperidone in real‐world clinical practice
Source: Neuropsychopharmacol Rep. 2024 Jul 8;44(3):614–9. doi: 10.1002/npr2.12459 (PMC11544453; doi:10.1002/npr2.12459)
Supplement: Supplementary file 2 — Figure S1. Figure S2. Figure S3. Figure S4. Figure S5. Figure S6. [file NPR2-44-614-s003.pptx]

## Slide 1
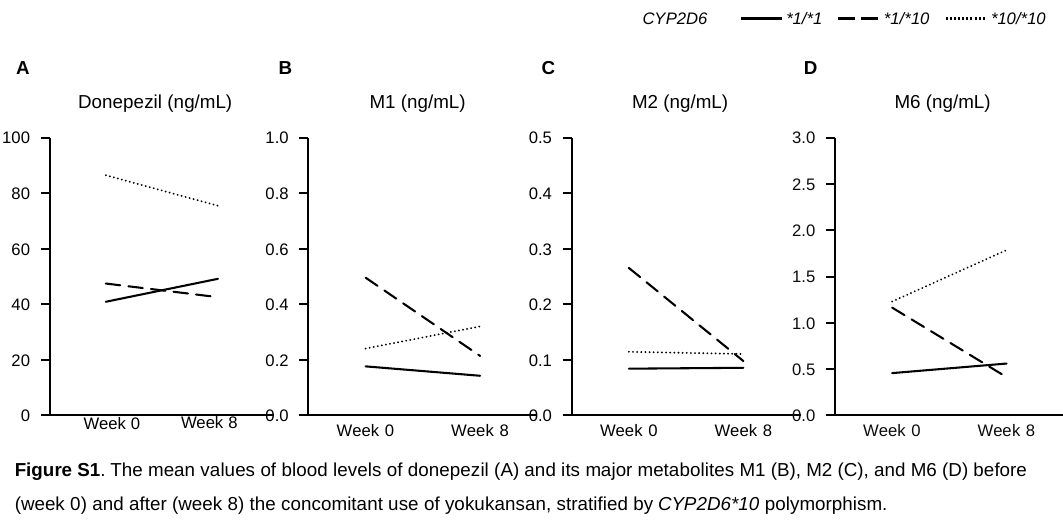

CYP2D6
*1/*1 *1/*10 *10/*10
A
B
C
D
Donepezil (ng/mL)
M1 (ng/mL)
M2 (ng/mL)
M6 (ng/mL)
[unsupported chart]
[unsupported chart]
[unsupported chart]
[unsupported chart]
Week 8
Week 0
Figure S1. The mean values of blood levels of donepezil (A) and its major metabolites M1 (B), M2 (C), and M6 (D) before (week 0) and after (week 8) the concomitant use of yokukansan, stratified by CYP2D6*10 polymorphism.

## Slide 2
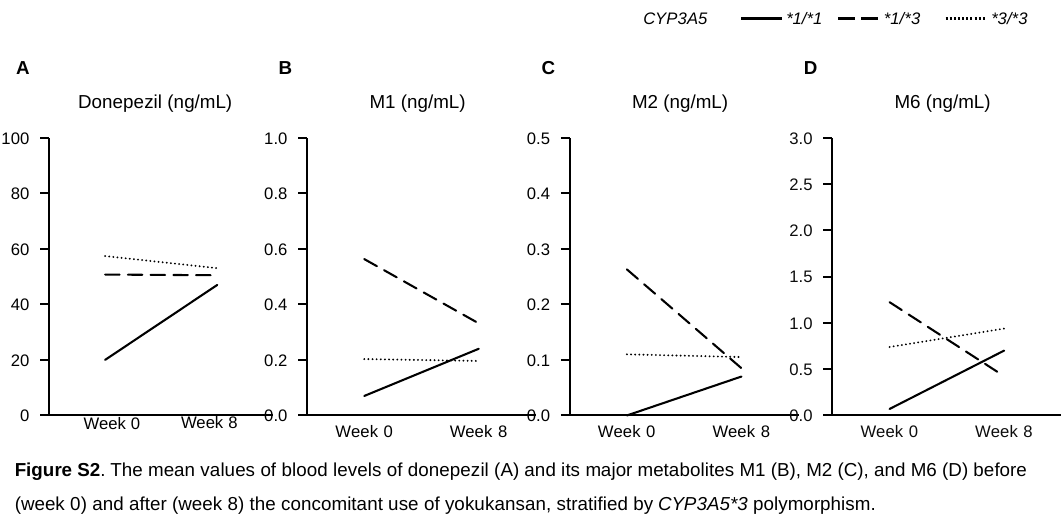

CYP3A5
*1/*1 *1/*3 *3/*3
A
B
C
D
Donepezil (ng/mL)
M1 (ng/mL)
M2 (ng/mL)
M6 (ng/mL)
[unsupported chart]
[unsupported chart]
[unsupported chart]
[unsupported chart]
Week 8
Week 0
Figure S2. The mean values of blood levels of donepezil (A) and its major metabolites M1 (B), M2 (C), and M6 (D) before (week 0) and after (week 8) the concomitant use of yokukansan, stratified by CYP3A5*3 polymorphism.

## Slide 3
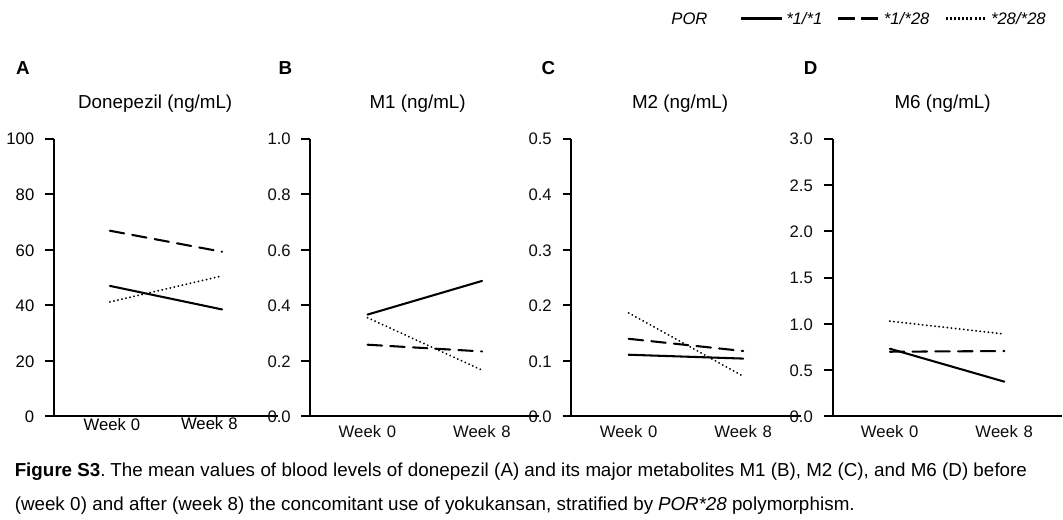

POR
*1/*1 *1/*28 *28/*28
A
B
C
D
Donepezil (ng/mL)
M1 (ng/mL)
M2 (ng/mL)
M6 (ng/mL)
[unsupported chart]
[unsupported chart]
[unsupported chart]
[unsupported chart]
Week 8
Week 0
Figure S3. The mean values of blood levels of donepezil (A) and its major metabolites M1 (B), M2 (C), and M6 (D) before (week 0) and after (week 8) the concomitant use of yokukansan, stratified by POR*28 polymorphism.

## Slide 4
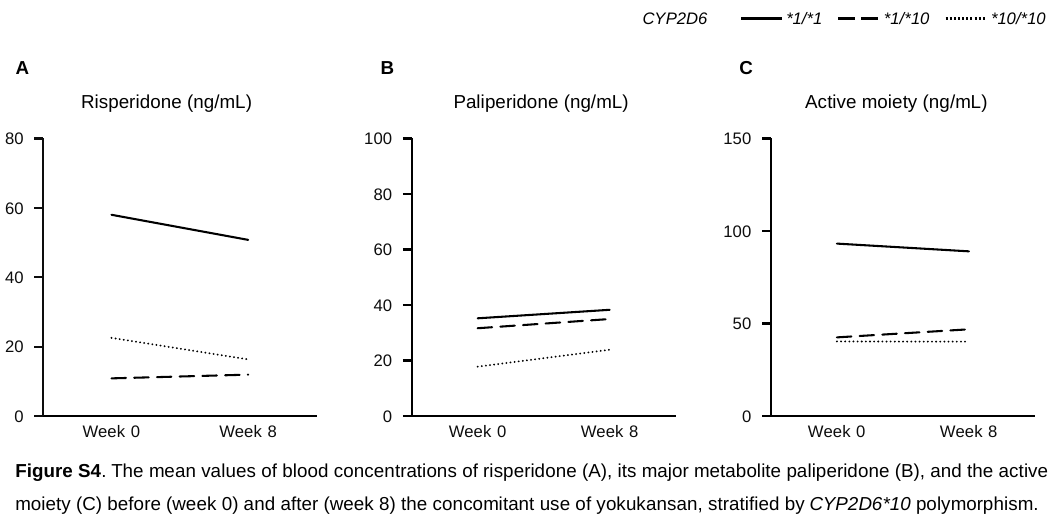

CYP2D6
*1/*1 *1/*10 *10/*10
A
B
C
Risperidone (ng/mL)
Paliperidone (ng/mL)
Active moiety (ng/mL)
### Chart
| Category | | | | |
|---|---|---|---|---|
| Week 0 | None | 58.008 | 10.86862499999999 | 22.53999999999998 |
| Week 8 | None | 50.772 | 11.94199999999999 | 16.332 |
### Chart
| Category | | | | |
|---|---|---|---|---|
| Week 0 | None | 35.21599999999999 | 31.63562499999999 | 17.784 |
| Week 8 | None | 38.28000000000002 | 34.95724999999998 | 23.90399999999998 |
### Chart
| Category | | | | |
|---|---|---|---|---|
| Week 0 | None | 93.22400000000003 | 42.50425000000001 | 40.32400000000001 |
| Week 8 | None | 89.05200000000004 | 46.89925000000002 | 40.23599999999997 |Figure S4. The mean values of blood concentrations of risperidone (A), its major metabolite paliperidone (B), and the active moiety (C) before (week 0) and after (week 8) the concomitant use of yokukansan, stratified by CYP2D6*10 polymorphism.

## Slide 5
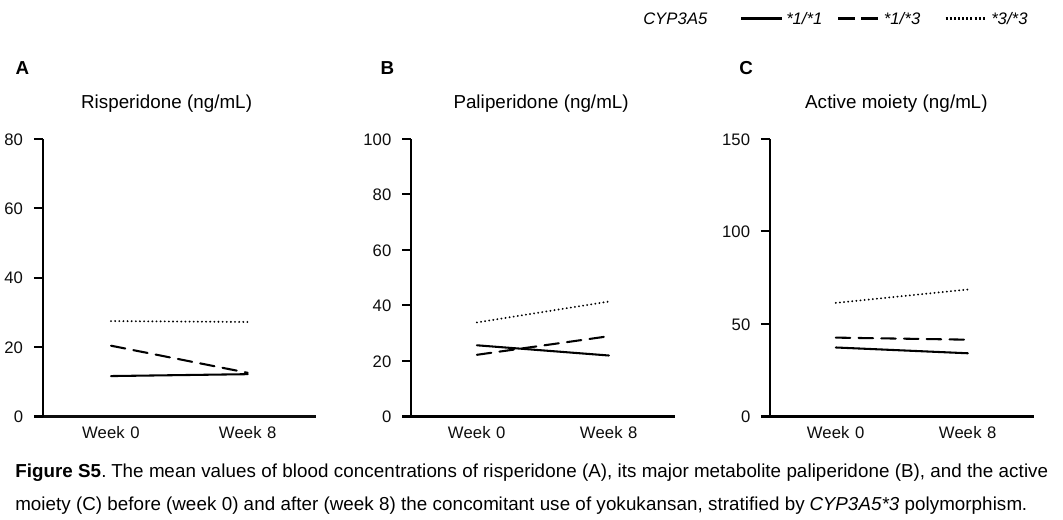

CYP3A5
*1/*1 *1/*3 *3/*3
A
B
C
Risperidone (ng/mL)
Paliperidone (ng/mL)
Active moiety (ng/mL)
### Chart
| Category | | | | |
|---|---|---|---|---|
| Week 0 | None | 11.65299999999999 | 20.39599999999999 | 27.48816666666666 |
| Week 8 | None | 12.19149999999999 | 12.576 | 27.22299999999998 |
### Chart
| Category | | | | |
|---|---|---|---|---|
| Week 0 | None | 25.64050000000001 | 22.20799999999999 | 33.87649999999999 |
| Week 8 | None | 21.96749999999999 | 28.90600000000001 | 41.38999999999997 |
### Chart
| Category | | | | |
|---|---|---|---|---|
| Week 0 | None | 37.29349999999999 | 42.60400000000002 | 61.36466666666669 |
| Week 8 | None | 34.15900000000001 | 41.48200000000003 | 68.61299999999999 |Figure S5. The mean values of blood concentrations of risperidone (A), its major metabolite paliperidone (B), and the active moiety (C) before (week 0) and after (week 8) the concomitant use of yokukansan, stratified by CYP3A5*3 polymorphism.

## Slide 6
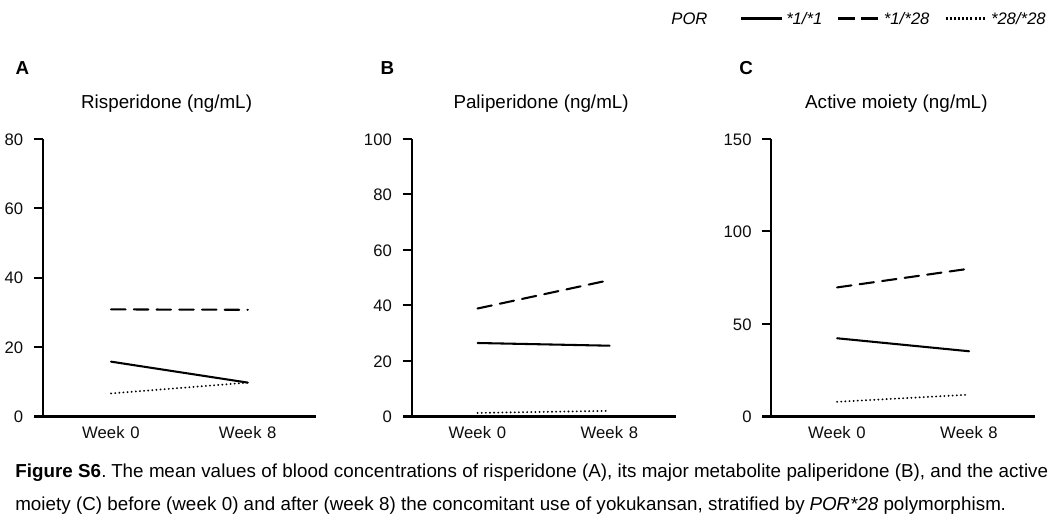

POR
*1/*1 *1/*28 *28/*28
A
B
C
Risperidone (ng/mL)
Paliperidone (ng/mL)
Active moiety (ng/mL)
### Chart
| Category | | | | |
|---|---|---|---|---|
| Week 0 | None | 15.79133333333333 | 30.85083333333333 | 6.635999999999992 |
| Week 8 | None | 9.751666666666662 | 30.72833333333334 | 9.763999999999951 |
### Chart
| Category | | | | |
|---|---|---|---|---|
| Week 0 | None | 26.46033333333331 | 38.88316666666666 | 1.296000000000011 |
| Week 8 | None | 25.47966666666665 | 49.14866666666667 | 2.031999999999976 |
### Chart
| Category | | | | |
|---|---|---|---|---|
| Week 0 | None | 42.25166666666668 | 69.73400000000001 | 7.932000000000034 |
| Week 8 | None | 35.23133333333335 | 79.87700000000002 | 11.79600000000002 |Figure S6. The mean values of blood concentrations of risperidone (A), its major metabolite paliperidone (B), and the active moiety (C) before (week 0) and after (week 8) the concomitant use of yokukansan, stratified by POR*28 polymorphism.
