# Supplementary material for: Effect of concomitant use of yokukansan on steady‐state blood concentrations of donepezil and risperidone in real‐world clinical practice
Source: Neuropsychopharmacol Rep. 2024 Jul 8;44(3):614–9. doi: 10.1002/npr2.12459 (PMC11544453; doi:10.1002/npr2.12459)
Supplement: Supplementary file 3 — Table S1. Table S2. Table S3. [file NPR2-44-614-s001.docx]

**Table S1**. Characteristics of study subjects at before (week 0) the concomitant use of yokukansan.

|  | **Patients treated with donepezil** | **Patients treated with risperidone** |
| --- | --- | --- |
| Disorder | Dementia | Schizophrenia |
| Number of subjects | 15 | 8 |
| Male/Female | 4 / 11 | 3 / 5 |
| Age (years) | 79.1 ± 7.2 | 52.5 ± 16.1 |
| Weight (kg) | 48.6 ± 7.9 | 69.9 ± 13.3 |
| Hight (cm) | 152.9 ± 6.4 | 162.1 ± 5.3 |
| Daily dose of donepezil or risperidone | 5 mg: 15 | 1 mg: 4 / 5 mg: 3 / 8 mg: 1 |
| Current vs. Past vs. Never smokers | 1 / 1 / 13 | 2 / 0 / 6 |
| *CYP2D6* *non-*5/non-*5* vs. *non-*5/*5* vs. **5/*5* | 11 / 0 / 0 | 8 / 0 / 0 |
| *CYP2D6* *non-*10/non-*10* vs. *non-*10/*10* vs. **10/*10* | 6 / 7 / 2 | 1 / 5 / 2 |
| *CYP3A5* *non-*3/non-*3* vs. *non-*3/*3* vs. **3/*3* | 1 / 4 / 10 | 2 / 2 / 4 |
| *POR* *non-*28/non-*28* vs. *non-*28/*28* vs. **28/*28* | 2 / 8 / 5 | 3 / 4 / 1 |

Values are means ± standard deviation or number of subjects. CYP, cytochrome P450; POR, P450 oxidoreductase

**Table S2**. The effect of genotype interaction with and without the concomitant use of yokukansan (week 0 vs. week 8) on blood levels of donepezil and its major metabolites M1, M2, and M6.

| Objective variable | Factor | *P-*value^†^ | *P*-value of interaction^†^  (i.e., week × genotype) |
| --- | --- | --- | --- |
| Donepezil conc. (ng/mL) | Week (i.e., week 0 vs. week 8) | 0.608 | 0.308 |
|  | *CYP2D6*10* genotype | 0.131 |  |
|  | *non-*10/non-*10* vs. *non-*10/*10* vs. **10/*10* |  |  |
| Donepezil conc. (ng/mL) | Week (i.e., week 0 vs. week 8) | 0.410 | 0.398 |
|  | *CYP3A5*3* genotype | 0.614 |  |
|  | *non-*3/non-*3* vs. *non-*3/*3* vs. **3/*3* |  |  |
| Donepezil conc. (ng/mL) | Week (i.e., week 0 vs. week 8) | 0.769 | 0.408 |
|  | *POR*28* genotype | 0.401 |  |
|  | *non-*28/non-*28* vs. *non-*28/*28* vs. **28/*28* |  |  |
| M1 conc. (ng/mL) | Week (i.e., week 0 vs. week 8) | 0.700 | 0.451 |
|  | *CYP2D6*10* genotype | 0.573 |  |
|  | *non-*10/non-*10* vs. *non-*10/*10* vs. **10/*10* |  |  |
| M1 conc. (ng/mL) | Week (i.e., week 0 vs. week 8) | 0.881 | 0.366 |
|  | *CYP3A5*3* genotype | 0.126 |  |
|  | *non-*3/non-*3* vs. *non-*3/*3* vs. **3/*3* |  |  |
| M1 conc. (ng/mL) | Week (i.e., week 0 vs. week 8) | 0.821 | 0.440 |
|  | *POR*28* genotype | 0.560 |  |
|  | *non-*28/non-*28* vs. *non-*28/*28* vs. **28/*28* |  |  |
| M2 conc. (ng/mL) | Week (i.e., week 0 vs. week 8) | 0.543 | 0.566 |
|  | *CYP2D6*10* genotype | 0.343 |  |
|  | *non-*10/non-*10* vs. *non-*10/*10* vs. **10/*10* |  |  |
| M2 conc. (ng/mL) | Week (i.e., week 0 vs. week 8) | 0.570 | 0.216 |
|  | *CYP3A5*3* genotype | 0.400 |  |
|  | *non-*3/non-*3* vs. *non-*3/*3* vs. **3/*3* |  |  |
| M2 conc. (ng/mL) | Week (i.e., week 0 vs. week 8) | 0.432 | 0.619 |
|  | *POR*28* genotype | 0.936 |  |
|  | *non-*28/non-*28* vs. *non-*28/*28* vs. **28/*28* |  |  |
| M6 conc. (ng/mL) | Week (i.e., week 0 vs. week 8) | 0.720 | 0.352 |
|  | *CYP2D6*10* genotype | 0.032 |  |
|  | *non-*10/non-*10* vs. *non-*10/*10* vs. **10/*10* |  |  |
| M6 conc. (ng/mL) | Week (i.e., week 0 vs. week 8) | 0.999 | 0.139 |
|  | *CYP3A5*3* genotype | 0.740 |  |
|  | *non-*3/non-*3* vs. *non-*3/*3* vs. **3/*3* |  |  |
| M6 conc. (ng/mL) | Week (i.e., week 0 vs. week 8) | 0.634 | 0.835 |
|  | *POR*28* genotype | 0.574 |  |
|  | *non-*28/non-*28* vs. *non-*28/*28* vs. **28/*28* |  |  |

^†^Repeated measures analysis of variance

**Table S3**. The effect of genotype interaction with and without the concomitant use of yokukansan (week 0 vs. week 8) on blood levels of risperidone, its major metabolite paliperidone, and active moiety.

| Objective variable | Factor | *P-*value^†^ | *P*-value of interaction^†^  (i.e., week × genotype) |
| --- | --- | --- | --- |
| Risperidone conc. (ng/mL) | Week (i.e., week 0 vs. week 8) | 0.291 | 0.320 |
|  | *CYP2D6*10* genotype | 0.172 |  |
|  | *non-*10/non-*10* vs. *non-*10/*10* vs. **10/*10* |  |  |
| Risperidone conc. (ng/mL) | Week (i.e., week 0 vs. week 8) | 0.719 | 0.387 |
|  | *CYP3A5*3* genotype | 0.893 |  |
|  | *non-*3/non-*3* vs. *non-*3/*3* vs. **3/*3* |  |  |
| Risperidone conc. (ng/mL) | Week (i.e., week 0 vs. week 8) | 0.698 | 0.395 |
|  | *POR*28* genotype | 0.669 |  |
|  | *non-*28/non-*28* vs. *non-*28/*28* vs. **28/*28* |  |  |
| Paliperidone conc. (ng/mL) | Week (i.e., week 0 vs. week 8) | 0.425 | 0.876 |
|  | *CYP2D6*10* genotype | 0.859 |  |
|  | *non-*10/non-*10* vs. *non-*10/*10* vs. **10/*10* |  |  |
| Paliperidone conc. (ng/mL) | Week (i.e., week 0 vs. week 8) | 0.350 | 0.975 |
|  | *CYP3A5*3* genotype | 0.838 |  |
|  | *non-*3/non-*3* vs. *non-*3/*3* vs. **3/*3* |  |  |
| Paliperidone conc. (ng/mL) | Week (i.e., week 0 vs. week 8) | 0.449 | 0.830 |
|  | *POR*28* genotype | 0.610 |  |
|  | *non-*28/non-*28* vs. *non-*28/*28* vs. **28/*28* |  |  |
| Active moiety conc. (ng/mL) | Week (i.e., week 0 vs. week 8) | 0.700 | 0.577 |
|  | *CYP2D6*10* genotype | 0.815 |  |
|  | *non-*10/non-*10* vs. *non-*10/*10* vs. **10/*10* |  |  |
| Active moiety conc. (ng/mL) | Week (i.e., week 0 vs. week 8) | 0.454 | 0.779 |
|  | *CYP3A5*3* genotype | 0.823 |  |
|  | *non-*3/non-*3* vs. *non-*3/*3* vs. **3/*3* |  |  |
| Active moiety conc. (ng/mL) | Week (i.e., week 0 vs. week 8) | 0.581 | 0.718 |
|  | *POR*28* genotype | 0.545 |  |
|  | *non-*28/non-*28* vs. *non-*28/*28* vs. **28/*28* |  |  |

^†^Repeated measures analysis of variance
